# Idiographic Dynamics between Suicide Ideation and Depression, Anxiety and Posttraumatic Stress Symptoms in Persons Living with HIV: A Pilot Study

**DOI:** 10.17505/jpor.2022.24856

**Published:** 2022-12-22

**Authors:** Yiqin Zhu, Thomas Rodebaugh, Kevin Narine, Lily A. Brown

**Affiliations:** 1 Department of Psychiatry, University of Pennsylvania; 2 Department of Psychological and Brain Science, Washington University at St. Louis; 3 Department of Clinical Psychology, William James College

**Keywords:** Suicide, HIV, anxiety, depression, PTSD, idiographic models

## Abstract

Background: Given that suicide ideation (SI) fluctuates drastically over short periods of time and is heterogenous across individuals, idiographic suicide research is warranted. In this pilot study, we used intensive ecological momentary assessment (EMA) to examine whether anxiety, depression, and PTSD symptoms on a given day predicted next-day SI on a person-to-person basis. Methods: PLWH (*N* = 10) with past-month SI completed daily randomly assessed ratings of suicidal urges using the Suicide-Visual Analogue Scale (S-VAS) and daily assessed ratings of anxiety, depression, and PTSD symptoms for 28 days. We used N = 1 Dynamic Structural Equation Modeling to test whether depression, anxiety or PTSD symptoms in the prior day predicted next-day S-VAS for each individual. Results: Across all participants, S-VAS on a given day was not predicted by prior-day anxiety, PTSD symptoms or S-VAS. In one participant, higher depression symptoms predicted lower next-day S-VAS. Conclusions: Daily-level data may be insufficient to predict near-term increases in suicide risk based on anxiety, depression, or PTSD symptoms in PLWH. These findings suggest the importance of finer-grained assessments (e.g., assessing suicide risk and its correlates multiple times per day) to better understand changes in suicide risk over time among PLWH.

**Highlights:**

- Prior-day suicide urges did not predict next-day urges within an individual
- In one subject, higher depression predicted lower next-day suicide urges
- Simulation showed over 60 within-person data points are needed to detect medium effects

## Introduction

Persons living with HIV (PLWH) have higher suicide ideation and attempt rates than the general population (Badiee et al., 2012). About 21%, 5%, and 1-2% of PLWH report suicidal ideation (Ferlatte et al., 2017) or past-year suicide attempts, or die by suicide, respectively (Gurm et al., 2015); these rates are markedly higher than in the general population (4.8%, 0.6%, and 0.01%, respectively; Hedegaard & Warner, 2021). Given the elevated risk for suicide among PLWH, it is essential to understand predictors of suicidal ideation and behavior in this sample.

There are two significant challenges in predicting suicide. First, suicide ideation (SI) dramatically fluctuates over time (Kleiman et al., 2017; Rudd, 2006). It is critical to improve suicide prevention and treatment by using a longitudinal, real-time design (Franklin et al., 2017). Ecological momentary assessment (EMA) methods could benefit suicide research by reducing recall bias and improving accuracy (Gratch et al., 2021; Davidson et al., 2017).

Second, the frequency, duration, and intensity of the precipitants of suicide are highly heterogeneous (Bryan & Rudd, 2015; Huang et al., 2017) and can be influenced by different causal pathways. A recent meta-analysis showed significant within-individual relationships between negative affect and SI (Kuehn et al., 2022a). Pooled results over 13 datasets suggested that negative affect was 0.11 standard deviations higher before, relative to moments not followed by suicidal thinking (β = 0.11, 95% CI 0.03-0.19). However, the effect was small, and six datasets (46.2%) did not support this effect (either the effect was null or reversed). The mixed findings further highlight heterogeneity in the within-individual association between SI and negative affect, a generally wellestablished predictor of SI.

Given the heterogeneous nature of suicide, it is essential to understand suicide at the individual level. Yet, most studies rely on group-level effects and assume that a single pattern can apply to everyone in the group. The most common approaches that consider individual heterogeneity in suicide research are multilevel models with random effects (e.g., Husky et al., 2017; Kaurin et al., 2020; Wolford-Clevenger et al., 2020). Although such models consider individual deviances in magnitude from the average/fixed group-level effects, they still assumed the same pattern of effects across individuals in the group. Even including a covariate to account for phenomenological variance (such as average intensity or frequency), the models still consider consistent covariate effects on every person. As pointed out by Kuehn et al. (2022a) in their recent meta-analysis, ignoring within-person processes could run the risk of committing Simpson’s paradox (Simpson, 1951), an ecological fallacy in which between-person conclusions (e.g., those high in negative affect are more likely to experience SI) are expected to generalize to a within-person process (e.g., if someone experiences elevated negative affect, they then are more likely to experience SI).

Idiographic approaches, which collect intensive longitudinal data from one individual and then develop a personalized statistical model (e.g., Fisher at al., 2017; Kaurin et al., 2022; Piccirillo & Rodebaugh, 2022), hold the potential to improve suicide prediction. Such approaches were also in accordance with the Fluid Vulnerability Theory of Suicide (Bryan et al., 2020), which conceptualizes suicide as an inherently dynamic construct that follows a nonlinear time course and differs drastically within an individual over time. To our knowledge, only two published studies have used full idiographic methods to predict suicide ideation. Both studies found that the relationship between suicidal urges and risk factors varies markedly across individuals. In one study (Kuehn et al., 2022b), three adolescent inpatients discharged following a suicide attempt were intensively assessed over 28 days and provided data once a day on their suicide ideation and coping behaviors; the association between suicidal ideation and coping differed across the three individuals. However, the study did not provide power analysis. Therefore, it is unclear whether the traditional intensive longitudinal assessment schedule in suicide research (e.g., once a day for 28 days) provides sufficient data for full idiographic models or whether observations are needed to make inferences about the true associations.

In the other study (Kaurin et al., 2022), 153 individuals with borderline personality disorder were intensively assessed over 21 days and answered six random surveys per day. There was significant heterogeneity in risk factors for suicidal ideation, with few shared features among participants. Negative affect was a poor predictor of suicidal ideation, and even autoregressive effects were reversed across some participants. For some participants, higher suicidal ideation on a given day predicted lower suicidal ideation the next day; for others, higher suicidal ideation the next day for other participants. It is not clear if these patterns of findings hold among patients recruited based on acute suicidal ideation or among PLWH. Moreover, about 40% (58 out of 153) of the sample did not reach the inclusion criteria (> 60 completed EMA queries and no zero variance in any variables). Such an exclusion rate is not surprising, as participants would have needed to answer more than two daily assessments of suicidal ideation for eligibility. Patients struggling with thoughts of suicide may be reluctant to report suicide urges once a day, let alone more than twice a day. Thus, there is a need to determine ideal assessment methods that balance accuracy with participant burden.

This study aimed to use intensive data to examine whether negative affect and suicidal ideation on a given day predicted next-day suicide ideation on a person-to-person basis in suicidal PLWH. We also conducted power analysis to test whether a traditional intensive longitudinal assessment schedule would provide sufficient power to detect in future research. Based on prior cross-sectional research (e.g., Brake et al., 2017; Casey et al., 2022) and meta-analytic results (Kuehn et al., 2022a), we hypothesized that, for most participants, internalizing symptoms on the prior day would predict the next-day suicide ideation. Based on prior idiographic suicide research (Kuehn et al., 2022b; Kaurin et al., 2022), we hypothesized significant heterogeneity in the longitudinal associations across participants.

## Methods

### Participants

Participants (*N* = 10; 70% male; 60% Black; 50% identified as LGBTQ+) were HIV+ adults (Age: *M* = 53.0, *SD* = 11.6 years) who endorsed past month suicide ideation and had access to a smartphone. Exclusion criteria were an active psychotic illness or a past year manic episode. Participants were recruited through flyers placed at community health facilities.

### Measures

**Depression and anxiety**. Depression and anxiety moods were assessed using the EMA version (Moore et al., 2016) of the Patient-reported Outcomes measurement information (PROMIS) adult depression and anxiety short form instruments (Bjorner et al., 2014). We used three anxiety items and four depression items. Respondents were instructed to rate the frequency of their symptoms at the moment on a 5-point scale ranging from 1 (not at all) to 5 (very much). Total raw scores were calculated for depression and anxiety symptoms, respectively. Higher scores indicated higher symptom severity. The EMA version of the PROMIS short form showed good internal consistency in previous research (Anxiety: Cronbach’s *α* = 0.93; Depression: Cronbach’s *α* = 0.90; Moore et al., 2016) and strong sensitivity to change (Moore et al., 2016).

**Posttraumatic Stress Disorder (PTSD) symptoms**. PTSD symptoms were assessed by an abbreviated eight-item version of the Posttraumatic Stress Disorder Checklist— Specific (PCL-S; Weathers et al., 1993), derived from the Military Suicide Research Consortium Common Data Elements (Ringer et al., 2018). Respondents were instructed to rate the degree to which they were “bothered” by the problem or symptom in the past 24 hours on a 5-point Likert scale ranging from 1 (“not at all”) to 5 (“extremely”). A total symptom severity score was obtained by summing the scores from the eight items. The internal consistency of the eightitem PCL-M was excellent (Cronbach’s *α* = 0.94; Ringer et al., 2018).

**Suicide Urges**. Suicide urges (“urge to kill myself”) were assessed by the Suicide Visual Analog Scale (S-VAS; Bryan, 2019) on a horizontal sliding scale ranging from 0 (none) on the left anchor to 100 (extreme) on the right anchor. Initially, the S-VAS was presented with the slide indicator in the “none” position; participants were instructed to indicate their response by moving the slide indicator. The S-VAS has good convergent validity with other suicide urge measures and predictive validity for suicide attempts (Bryan, 2019).

### Procedures

Procedures were approved by the Institutional Review Board at the University of Pennsylvania. Following the phone screening that determined initial study eligibility, participants presented to the laboratory to complete informed consent and a comprehensive intake evaluation. During the evaluation, exclusion diagnoses (i.e., active psychosis and a past year manic episode) were determined using the MiniInternational Neuropsychiatric Interview (MINI; Sheehan et al., 1998). Participants were provided with $40 for completion of the baseline evaluation.

**Ecological Momentary Assessment Data Collection.** Upon completion of the evaluation, eligible participants downloaded MetricWire onto their smartphones to begin receiving EMA surveys the next day for 28 days. The depression, anxiety, and PTSD symptoms surveys were administered once a day at a random time. The S-VAS was administered four times daily for 28 days: twice at random periods at every hour of the waking day, once at awakening in the morning sleep diary, and once at night before sleep in the nighttime sleep diary. Each day, the morning sleep diary was activated at 6:00 AM, and the nighttime sleep diary was activated at 9:00 PM. Other surveys were delivered seven days a week between 8:00 AM and 11:00 PM. There was a fivehour completion window for the morning sleep diary (i.e., 6:00 AM to 11:00 AM), a two-hour completion window (i.e., 9:00 PM to 11:00 PM) for the nighttime sleep diary, and a one-hour completion window for other surveys. At the end of the 28-day assessment, they returned to the clinic for a final evaluation and were compensated $50 for the post-evaluation.

### Data Analytic Plan

**Data detrending**. Before all study analyses, each individual’s time series were detrended by taking the residuals of each variable (anxiety, depression, PTSD symptoms, and suicide urges) regressed on linear continuous time.

**Figure 1:**
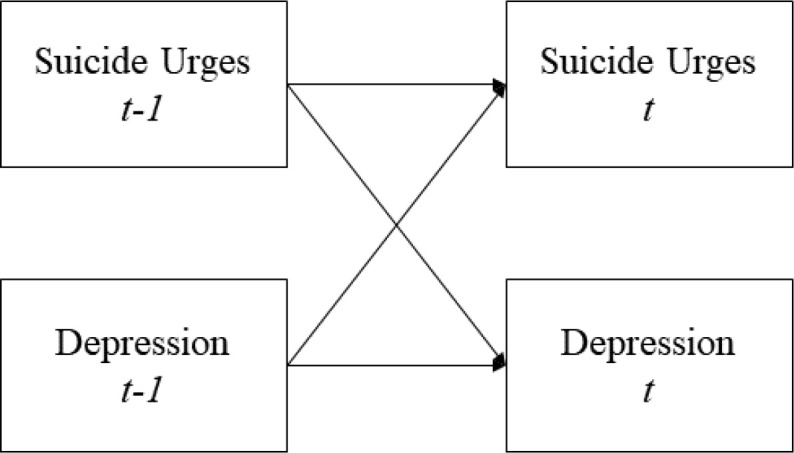
An example of our conceptualized lag-1 N=1 dynamic structural equation model. Error variances not shown for simplicity. Note that all time points in the data are included, although only two are depicted here.

***N* = 1 Dynamic Structural Equation Modeling (DSEM).** We ran three models for every individual. Each model includes the suicide urges and one psychopathology symptom variable (depression, anxiety, or PTSD symptoms). Taking the bivariate model between suicide urges and depression as an example (Figure 1), the model included cross-lagged paths between suicide urges and depression, auto-regressive paths of suicide urges, and auto-regressive paths of depression. This approach allowed us to examine individual differences in whether depression (or anxiety and PTSD symptoms) on the prior day predicted subsequent suicide urges. Significance was defined at *p* < 0.005 to adjust for familywise errors.

To construct *N*=1 DSEM models, an open lag-1 model was run without specifying iterations. A model is considered to meet the convergence criterion if it produced a low and stable proportional scale reduction (PSR) factor (a cutoff of 1.01 was used). After the model converged, successive models were run with twice as many iterations, and the PSR was evaluated. The model was considered to be final when two consecutive models converged with PSR lower than 1.01. When necessary, commands to prune iterations (Asparouhov, 2014) were used to achieve model convergence.

**Monte Carlo Simulation Analysis**. We considered the time points of assessment (up to 112 for suicide ideation, 28 for depression, anxiety, and PTSD symptoms) to be sufficient because such analysis typically has 20-200 time points (Muthén and Muthén, 1998-2017), but we were aware of a general consensus among experts that more time points might be preferable. We ran Monte Carlo Simulations with 1000 replications to examine the power of idiographic models. Power values of at least 80% are generally viewed as desirable.

We first simulated the power to detect the effect we observed using the number of observations in this study. Then we simulated an increase in the number of observations from 20 to 200 (step interval: 20) to examine how many valid observations will be needed for a desirable power (>80%) based on each individual’s observed effect size. We stopped the simulation when the power of a lagged path predicting next-day suicide urges first exceeds 80% or when the number of observations reaches 200 (because it represents a number challenging to obtain, and thus a pragmatic upper limit), whichever came first.

## Results

### Key Variables, Survey Response, and Contemporaneous Correlations

During the 28-day EMA period, participants completed an average of 57.9 observations for all suicide urges surveys (range: 26-104), an average of 18.0 observations for the anxiety and depression survey (range: 1-28), and an average of 17.9 observations for the PTSD symptoms survey (range: 1-27).

The means and standard deviations of each variable (calculated for each person) varied considerably (Table 1). Subject 9 was excluded for subsequent bi-variate analysis because of too few observations.

**Table 1:** Valid Observation Numbers, Means, and Standard Deviations for Suicide Urges, Depression, Anxiety, and PTSD Symptoms at the Individual Level

Subject ID	Suicide Urges (4 per day)			Anxiety			Depression			PTSD		
	N	M	SD	N	M	SD	N	M	SD	N	M	SD
1	101	19.6	13.5	22	6.5	2.3	22	6.8	2.0	22	19.1	4.1
2	78	8.9	5.3	19	8.1	1.4	19	11.4	1.5	20	23,1	2.7
3	40	5.3	11.3	16	6.8	2.5	16	6.4	2.0	15	13.6	4.9
4	30	2.3	6.6	13	7.7	2.0	13	10.7	1.6	14	26.3	2.4
5	33	2.3	4.0	16	7.3	2.2	16	5.3	2.8	15	15.1	2.7
6	54	0.3	2.1	28	4	1.6	28	5.2	1.7	27	3.5	9.5
7	70	6.8	5.0	24	8.8	2.2	24	13.4	1.4	20	18.9	2.5
8	104	61.2	7.8	27	15	0	27	19.8	0.8	27	36.5	1.3
9	26	10.1	10.1	1	10	NA	1	10	NA	1	20	NA
10	43	2.4	4.9	13	6.5	2.4	13	6.4	2.0	17	16.6	4.8

Bivariate contemporaneous correlations (i.e., correlations between each variable at the same time-point) between studied variables were calculated for each individual and showed substantial variability (Table 2).

**Table 2:** Summary Statistics for Within-person Contemporaneous Correlations of Study Variables

Variables	Suicide urges and Depression	Suicide urges and Anxiety	Suicide urges and PTSD	Depression and Anxiety	Anxiety and PTSD	Depression and PTSD
Range	-.18 - .66	.07 – .75	-.13 – .67	-.38 – .92	-.60 – .78	-.46 – .77
M	.30	.37	.30	.50	.27	.25
SD	.29	.24	.32	.39	.51	.41

*Note:* First, within-person correlations between each of the study variables were computed.

*M* = the nomothetic average of the within-person correlations for each pair of variables; *SD* = standard deviation.

### Idiographic Models

**Bivariate Model between suicide urges and depression**. Among the nine participants examined, all individual-level models reached the convergence criterion. One participant (11,1%, participant 8) had a model that demonstrated a significant cross-lagged effect of depression mood on next-day suicide urges (*β* = -.49, p = .005). For the remaining eight participants, models did not demonstrate any significant effect.

**Bivariate Model between suicide urges and anxiety**. Among the nine participants examined, 2 participants’ individual-level model did not reach the convergence criterion (ID=4, 8), and therefore the parameter estimates were not trustworthy. For the remaining seven participants, models did not demonstrate any significant effect. For the participants whose model converged, suicide urges were not predicted by higher anxiety symptoms in the prior day.

**Bivariate Model between suicide urges and PTSD.** Among the nine participants examined, all individual-level models reached the convergence criterion. Models did not demonstrate any significant effect. Suicide urges were not predicted by higher PTSD symptoms in the prior day for any participants (see Table 3).

**Table 3:** Standardized Path Coefficients for N=1 Bivariate DSEM Models for Each Individual

Bivariate Model	ID	Suicide urges (*t*)		Internalizing (*t*)	
		Suicide urges (*t*-1)	Internalizing^a^ (*t*-1)	Suicide urges (*t*-1)	Internalizing^a^ (*t*-1)
Suicide Urges & Depression	1 2 3 4 5 6 7 8 10	0.13 -0.21 -0.05 0.24 -0.02 -0.32 0.28 0.19 -0.09	0.23 -0.03 0.60 0.20 0.02 0.52 0.09 -0.49* -0.36	0.68 0.01 0.12 0.29 0.08 -0.84 0.00 -0.42 0.23	-0.09 -0.06 0.75 -0.25 -0.16 0.52 -0.04 -0.10 -0.47
	
Suicide Urges & Anxiety	1 2 3 4 5 6 7 8 10	0.06 -0.19 0.09 NA1 -0.03 -0.38 0.24 NA2 -0.22	0.31 -0.29 0.46 NA1 0.21 0.57 0.13 NA2 0.28	0.50 -0.31 0.34 NA1 -0.36 -0.33 -0.13 NA2 -0.20	-0.03 0.00 0.50 NA1 -0.15 0.18 -0.37 NA2 -0.56
	
Suicide Urges & PTSD	1 2 3 4 5 6 7 8 10	0.33 -0.29 0.20 0.37 0.00 -0.14 0.25 0.10 -0.28	0.21 -0.21 0.37 0.16 0.64 0.17 0.43 -0.14 -0.02	0.35 0.02 0.37 0.28 -0.40 0.11 0.01 -0.14 -0.28	0.06 -0.03 -0.12 -0.29 -0.09 0.60 -0.06 0.03 -0.07

*Note:* All statistics were calculated at the individual level. NA1: Model did not meet the convergence criterion (PSR < 1.01 for two consecutive models); NA2: One of the variables has a variance of 0, making the model impossible to run.

a Internalizing represents the variable used in a bivariate model (i.e., depression, anxiety, or PTSD), corresponding to the variable in the “Psychopathology Variable” column.

* *p* < 0.005

### Monte Carlo Simulation Analysis

For the two participants that had a model that demonstrated at least one statistically significant path (cross-lagged effect or autoregressive path) from depression to suicide urges, power suggested a reasonable, although still modest, ability to detect effects (.43 for participant 3, and .54 for participant 8). For models of other participants, the power was low. (See Table S1-S3 in Appendix 1 for the complete results of these simulations.) To have a desirable power (>80%) to detect at least one significant lagged path to predict suicide based on the effect sizes detected in this study, 100 valid observations would be needed for about two-thirds of participants (64%; 16 out of 25 models; Table 4). Considering an average completion rate of 51.8% for the suicide urges survey and 64.2% for internalizing symptoms survey based on the study, 156 to 193 prompts to collect suicide urges and their predictors would be needed.

**Table 4:** Monte Carlo Simulations – Number of Observations Needed for Power over 80% to Detect the Effects of Predicting Next-day Suicide Urges Observed in the Current Study

ID	Depression	Anxiety	PTSD
1	140	80	100
2	180	100	100
3	40	40	80
4	160	NA	80
5	200	160	40
6	40	40	200
7	120	160	60
8	40	NA	200
10	60	80	100

*Note:* NA: estimates not available because the participant’s model did not meet the convergence criteria and we do not have a reference to create population values for coefficients.

## Discussion

In a sample of suicidal PLWH who were intensively assessed for four weeks, suicidal ideation on a given day was not predicted by prior-day depression, anxiety, or PTSD symptoms. In one participant, next-day suicide urges were predicted by prior-day depression. This finding was not consistent with our hypotheses. Furthermore, suicidal ideation was not significantly predicted by prior-day suicidal ideation. Similar results were observed for lack of autoregressive effects on anxiety and PTSD, such that prior-day anxiety and PTSD did not predict next-day anxiety and PTSD, respectively. These findings highlight the difficulty in forecasting next-day suicidal ideation and negative affect in patients reporting baseline suicidal ideation even with moderately intensive longitudinal data.

We formed study hypotheses based on group-level findings in the general population and from PLWH specifically. Both cross-sectional studies (e.g., Brake et al., 2017; Casey et al., 2022) and non-intensive longitudinal studies (e.g., Ribeiro et al., 2018; Stanley et al., 2019) have demonstrated that PTSD, anxiety, and depression symptoms were each significantly associated increased risk for suicidal ideation. In prior studies, significant autoregressive effects are either assumed (Brown et al., 2019) (and therefore included as covariates in analyses) or supported empirically (e.g., Kleiman et al., 2017).

There are several possible reasons why our individual-level findings differ from group effects. First, as is raised by Fisher and colleagues (2018), within-individual relationships between variables could show different (even opposite) patterns from between-individual relationships. Indeed, although negative affect is a well-established risk factor in group-level studies, within-person prediction of negative affect on suicide urges (β = 0.11) was small according to metaanalysis (Kuehn et al., 2022a). Most prior research on daily changes in SI examined group-level effects (e.g., Husky et al., 2017; Littlewood et al., 2019). Only two studies examined the individual-level relationships between suicide and its risk factors (Kaurin et al., 2022; Kuehn et al., 2022b). Consistent with our findings, their models revealed high levels of heterogeneity in state risk factors related to suicidal ideation (Kaurin et al., 2022; Kuehn et al., 2022b), with no features shared among the majority of participants or even among relatively homogenous clusters of participants (Kaurin et al., 2022). Indeed, in many idiographic studies in different content areas, individual models commonly demonstrated very different model structures from group-level models (for substance use, see Ashlock et al., 2021; for mood and anxiety disorders, see Fisher et al., 2017, Piccirillo & Rodebaugh, 2022). Given that this is the first study to examine person-specific suicide predictions in PLWH using an intensive longitudinal design, more research is needed for this high-risk population.

Second, it might be difficult to predict daily changes in suicide urges, given the fact that suicide urges can vary dramatically over even short periods such as hours or days (e.g., Kleiman et al., 2017). In our prior research, we demonstrated significant variability in suicidal urges, even within a 24-hour period (Brown et al., in press). Consistent with prior studies (Ben-Zee et al., 2012; Kleiman et al., 2017), our study showed that established suicide risk factors might be less useful in predicting suicidal ideation on a daily basis. It is unclear what time scale SI can be best predicted by its risk factors. Suicidal ideation may be better characterized by non-linear, sudden changes, such as those observable using the CUSP Catastrophe Models (Bryan et al., 2020). However, these analyses would require much more frequent sampling than that needed by DSEM. Future research might benefit from more frequent observations (e.g., hour-by-hour) to try to better capture dynamic features of this rapidly unfolding process and determine the optimal time scale of suicide prediction. However, future research will need to balance participant fatigue with more frequent assessments, which may limit the number of assessments per day or days of assessments for feasibility. The results of our simulations can be helpful to researchers designing such studies because they provide guidance as to the number of time points that are likely to be needed to detect the effects that were observed here.

Prior affect symptoms levels did not precede and predict themselves for most participants. Such observation points to the instability of the affective state in daily life. This is consistent with prior studies that have shown anxiety symptoms were only partially stable at the within-person level (e.g., Groen et al., 2021; Murray et al., 2022). For example, in a Dutch general population sample (N = 767) who were assessed three times a day over 30 days, about 78% of participants did not show significant autoregressive effects on anxiety symptoms (Groen et al., 2021). Our result replicated the findings that affective risk factors were unstable at the individual level, which might partly explain the difficulty of suicide prediction over a short period of time. Notably, the stability of affect might be quite different in different populations; for example, we would expect notable stability of anxiety in a population of individuals with diagnosable anxiety disorders.

### Clinical Implications

This study has at least two key clinical implications. First, the study results support the argument that highly accurate real-world prediction of next-day suicidal ideation might be impossible without over 100 observations for at least many people living with HIV. As shown in the current EMA study, which assessed constructs on a day-by-day basis, suicide urges were not predicted by established predictors such as internalizing symptoms or prior-day suicidal ideation. Clinicians and researchers might need to reconsider the utility of attempting to predict suicide and instead focus their efforts on understanding suicide (Klonsky et al., 2020) or on deploying universal suicide mitigation strategies (e.g., Safety Planning, Stanley & Brown, 2012; Crisis Response Planning, Bryan, et al., 2017). It is possible, however, that the dynamic relationships between suicide urges and their predictors might play out with more observation points and a smaller time scale, such as on an hour-by-hour (or even minute-by-minute) basis. The optimal timescale of suicide prediction is still unclear. Second, given how the temporal relationships between suicide urges and their predictors varied across individuals, the study points to the potential importance of person-specific suicide risk assessment and prevention, and the study framework can be used to provide actionable information to formulate personalized interventions.

### Limitations

The present research had several limitations. First, although the current sample of PLWH was diverse regarding race/ethnicity/sexual/gender identity, this is a very small (*N* = 10) convenience sample, which increases the risk of bias. Moreover, whereas the current study period was designed for 28 days of assessments to follow previous suicide EMA designs, the number of observations did not have sufficient power to detect small effects as observed in the current study, according to the simulation results. The power of detecting important effects is not high, and could have prevented the detection of a real effect and biased estimates. According to the simulation results, to detect medium effects (DSEM path coefficient = 0.5), 60 to 70 observation points (considering a compliance rate of 60%) would be needed. Since collecting data from people with active SI over 60 days may be less feasible than the current design of 28 days, future studies should consider more frequent assessments (e.g., 2 to 3 times a day for a consecutive month) to increase the power to detect effects. For example, with the current study design that collected suicide urges rating using a single item four times a day, 40% of participants had more than 70 valid data points (70, 78, 101, 104, respectively). However, negative affect was only assessed once per day, preventing finer-grained analyses of fluctuations in negative affect as a predictor of suicidal ideation over hours in a day. Third, a very large number of significance tests were calculated. Fourth, all constructs in the current report were measured using self-report. Future research might benefit from using passive assessment or objective indicators (e.g., physiological processes) to assess longitudinal predictors of suicide. Finally, the current study only examined internalizing symptoms as longitudinal predictors of suicide. There are also other risk factors that could predict suicide urges but were not assessed in the current study, such as hopelessness, shame, or guilt. The withinperson temporal relationships between these risk factors and suicide urge warrant future research examinations.

### Conclusion

In summary, in this sample of PLWH, negative affect was generally not predictive of next-day suicide ideation using an intensive longitudinal approach. Suicidal ideation was also not consistently predicted by prior day suicide ideation over the course of 28 days. This work suggests that suicide ideation might not be best predicted on a daily basis, and more frequent data with bigger sample size is needed to determine the optimal timescale of suicide prediction. The simulations we presented should be helpful for researchers pursuing such a goal.

## Data Availability

On request.

## Action editor

Lars-Gunnar Lundh served as action editor for this article.

## Appedendix 1

### Supplementary Materials

**Table S1:** Monte Carlo Simulations of Depression Symptoms Predicting Next-day Suicide Ideation – the Power to Detect the Effect That We Observed and corresponding Number of Observations

ID	N obs for each variable	Suicide urges (*t*)		Depression (*t)*	
		Suicide urges (*t-1*)	Depression (*t-1*)	Suicide urges (*t-1*)	Depression (*t-1*)
1	**Current Study Nobs**	**0.10**	**0.16**	**0.47**	**0.04**
	20	0.03	0.06	0.60	0.04
	40	0.07	0.23	0.95	0.09
	60	0.10	0.41	1.00	0.12
	80	0.15	0.56	1.00	0.17
	100	0.19	0.69	1.00	0.20
	120	0.23	0.78	1.00	0.23
	**140**	**0.29**	**0.86**	**1.00**	**0.28**
	160	0.31	0.90	1.00	0.29
	180	0.35	0.93	1.00	0.34
	200	0.39	0.95	1.00	0.37
2	**Current Study Nobs**	**0.22**	**0.05**	**0.04**	**0.01**
	20	0.08	0.03	0.03	0.02
	40	0.22	0.05	0.04	0.05
	60	0.34	0.05	0.04	0.07
	80	0.42	0.05	0.07	0.09
	100	0.51	0.05	0.05	0.08
	120	0.59	0.06	0.05	0.09
	140	0.68	0.06	0.04	0.11
	160	0.75	0.06	0.05	0.11
	**180**	**0.80**	**0.06**	**0.05**	**0.13**
	200	0.83	0.07	0.05	0.14
3	**Current Study Nobs**	**0.03**	**0.43**	**0.06**	**0.19**
	20	0.02	0.65	0.03	0.65
	**40**	**0.05**	**0.98**	**0.09**	**0.99**
	60	0.06	1.00	0.13	1.00
	80	0.08	1.00	0.17	1.00
	100	0.07	1.00	0.22	1.00
	120	0.09	1.00	0.27	1.00
	140	0.10	1.00	0.30	1.00
	160	0.10	1.00	0.34	1.00
	180	0.10	1.00	0.42	1.00
	200	0.13	1.00	0.45	1.00
4	**Current Study Nobs**	**0.03**	**0.04**	**0.03**	**0.01**
	20	0.05	0.05	0.13	0.13
	40	0.20	0.16	0.38	0.36
	60	0.34	0.29	0.56	0.52
	80	0.53	0.38	0.71	0.64
	100	0.63	0.51	0.79	0.74
	120	0.73	0.59	0.87	0.82
	140	0.77	0.69	0.93	0.87
	**160**	**0.84**	**0.74**	**0.96**	**0.91**
	180	0.89	0.80	0.98	0.95
	200	0.92	0.85	0.98	0.95
5	**Current Study Nobs**	**0.03**	**0.03**	**0.04**	**0.01**
	20	0.02	0.02	0.04	0.06
	40	0.03	0.03	0.06	0.15
	60	0.05	0.04	0.07	0.23
	80	0.04	0.04	0.10	0.30
	100	0.05	0.05	0.10	0.34
	120	0.05	0.05	0.13	0.41
	140	0.05	0.05	0.13	0.47
	160	0.06	0.05	0.15	0.50
	180	0.06	0.05	0.17	0.57
	**200**	**0.05**	**0.06**	**0.18**	**0.63**
6	**Current Study Nobs**	**0.29**	**0.77**	**0.79**	**0.30**
	20	0.23	0.706	0.887	0.559
	**40**	**0.59**	**0.99**	**1.00**	**0.95**
	60	0.81	1.00	1.00	1.00
	80	0.91	1.00	1.00	1.00
	100	0.95	1.00	1.00	1.00
	120	0.98	1.00	1.00	1.00
	140	0.99	1.00	1.00	1.00
	160	0.99	1.00	1.00	1.00
	180	1.00	1.00	1.00	1.00
	200	1.00	1.00	1.00	1.00
7	**Current Study Nobs**	**0.23**	**0.06**	**0.04**	**0.03**
	20	0.06	0.03	0.03	0.03
	40	0.26	0.05	0.04	0.05
	60	0.44	0.08	0.05	0.07
	80	0.63	0.10	0.05	0.08
	100	0.75	0.12	0.05	0.07
	**120**	**0.85**	**0.13**	**0.05**	**0.08**
	140	0.88	0.17	0.04	0.09
	160	0.91	0.17	0.04	0.09
	180	0.96	0.19	0.05	0.10
	200	0.97	0.21	0.05	0.11
8	**Current Study Nobs**	**0.25**	**0.54**	**0.37**	**0.05**
	20	0.030	0.34	0.23	0.05
	**40**	**0.16**	**0.84**	**0.69**	**0.11**
	60	0.27	0.97	0.91	0.17
	80	0.41	0.99	0.97	0.19
	100	0.53	1.00	0.99	0.21
	120	0.619	1.00	1.00	0.26
	140	0.667	1.00	1.00	0.27
	160	0.749	1.00	1.00	0.30
	180	0.796	1.00	1.00	0.34
	200	0.844	1.00	1.00	0.38
10	**Current Study Nobs**	**0.01**	**0.08**	**0.05**	**0.02**
	20	0.02	0.27	0.12	0.44
	40	0.06	0.66	0.30	0.84
	**60**	**0.08**	**0.85**	**0.45**	**0.94**
	80	0.12	0.93	0.56	0.99
	100	0.15	0.98	0.65	1.00
	120	0.17	0.99	0.73	1.00
	140	0.19	1.00	0.80	1.00
	160	0.19	1.00	0.83	1.00
	180	0.22	1.00	0.87	1.00
	200	0.26	1.00	0.92	1.00

**Table S2:** Monte Carlo Simulations of Anxiety Symptoms Predicting Next-day Suicide Ideation – the Power to Detect the Effect That We Observed and corresponding Number of Observations

ID	N obs for each variable	Suicide urges (*t*)		Anxiety (*t)*	
		Suicide urges (*t-1*)	Anxiety (*t-1*)	Suicide urges (*t-1*)	Anxiety (*t-1*)
1	**Current Study Nobs**	**0.04**	**0.23**	**0.31**	**0.02**
	20	0.02	0.12	0.34	0.02
	40	0.04	0.41	0.80	0.04
	60	0.04	0.64	0.95	0.05
	**80**	**0.05**	**0.81**	**0.99**	**0.06**
	100	0.07	0.89	1.00	0.06
	120	0.09	0.94	1.00	0.08
	140	0.10	0.97	1.00	0.08
	160	0.11	0.98	1.00	0.08
	180	0.10	1.00	1.00	0.07
	200	0.12	1.00	1.00	0.09
2	**Current Study Nobs**	**0.20**	**0.14**	**0.10**	**0.01**
	20	0.07	0.12	0.11	0.01
	40	0.20	0.39	0.38	0.04
	60	0.31	0.59	0.60	0.05
	80	0.41	0.73	0.76	0.04
	**100**	**0.49**	**0.84**	**0.86**	**0.04**
	120	0.55	0.91	0.92	0.04
	140	0.64	0.93	0.96	0.05
	160	0.70	0.96	0.98	0.06
	180	0.76	0.98	0.98	0.05
	200	0.80	0.99	1.00	0.06
3	**Current Study Nobs**	**0.04**	**0.20**	**0.08**	**0.04**
	20	0.02	0.34	0.17	0.26
	**40**	**0.04**	**0.81**	**0.49**	**0.81**
	60	0.06	0.96	0.75	0.96
	80	0.08	0.99	0.86	0.99
	100	0.11	1.00	0.93	1.00
	120	0.14	1.00	0.97	1.00
	140	0.16	1.00	0.99	1.00
	160	0.19	1.00	0.99	1.00
	180	0.19	1.00	1.00	1.00
	200	0.22	1.00	1.00	1.00
4	NA	NA	NA	NA	NA
5	**Current Study Nobs**	**0.03**	**0.09**	**0.10**	**0.02**
	20	0.02	0.10	0.21	0.06
	40	0.05	0.24	0.57	0.16
	60	0.05	0.36	0.78	0.24
	80	0.05	0.48	0.88	0.30
	100	0.05	0.61	0.93	0.37
	120	0.05	0.68	0.97	0.41
	140	0.07	0.78	0.99	0.47
	**160**	**0.07**	**0.81**	**1.00**	**0.50**
	180	0.07	0.86	1.00	0.57
	200	0.07	0.89	1.00	0.62
6	**Current Study Nobs**	**0.38**	**0.47**	**0.31**	**0.04**
	20	0.33	0.54	0.26	0.04
	**40**	**0.74**	**0.94**	**0.65**	**0.14**
	60	0.90	0.99	0.84	0.27
	80	0.97	1.00	0.93	0.36
	100	0.99	1.00	0.96	0.44
	120	0.99	1.00	0.99	0.53
	140	1.00	1.00	1.00	0.59
	160	1.00	1.00	1.00	0.67
	180	1.00	1.00	1.00	0.70
	200	1.00	1.00	1.00	0.77
7	**Current Study Nobs**	**0.17**	**0.07**	**0.06**	**0.08**
	20	0.05	0.04	0.04	0.24
	40	0.21	0.08	0.11	0.63
	60	0.35	0.15	0.16	0.79
	80	0.52	0.20	0.21	0.91
	100	0.64	0.26	0.27	0.96
	120	0.73	0.30	0.31	0.98
	140	0.78	0.35	0.36	0.99
	**160**	**0.84**	**0.42**	**0.41**	**1.00**
	180	0.89	0.43	0.46	1.00
	200	0.92	0.47	0.49	1.00
8	NA	NA	NA	NA	NA
10	**Current Study Nobs**	**0.08**	**0.06**	**0.05**	**0.03**
	20	0.09	0.17	0.09	0.54
	40	0.26	0.47	0.21	0.94
	60	0.41	0.68	0.36	0.99
	**80**	**0.51**	**0.81**	**0.48**	**1.00**
	100	0.60	0.90	0.57	1.00
	120	0.69	0.94	0.65	1.00
	140	0.77	0.97	0.70	1.00
	160	0.83	0.98	0.78	1.00
	180	0.89	1.00	0.81	1.00
	200	0.90	1.00	0.87	1.00

*Note:* NA: estimates not available because the participant’s model did not meet the convergence criteria and we do not have a reference to create population values for coefficients;

**Table S3:** Monte Carlo Simulations of PTSD Symptoms Predicting Next-day Suicide Ideation – the Power to Detect the Effect That We Observed and corresponding Number of Observations

ID	N obs for each variable	Suicide urges (*t*)		PTSD (*t)*	
		Suicide urges (*t-1*)	PTSD (*t-1*)	Suicide urges (*t-1*)	PTSD (*t-1*)
1	**Current Study Nobs**	**0.57**	**0.10**	**0.19**	**0.02**
	20	0.07	0.06	0.19	0.01
	40	0.37	0.17	0.53	0.03
	60	0.62	0.30	0.75	0.04
	80	0.78	0.42	0.88	0.06
	**100**	**0.87**	**0.53**	**0.94**	**0.07**
	120	0.94	0.63	0.98	0.08
	140	0.97	0.73	0.99	0.09
	160	0.98	0.79	0.99	0.10
	180	0.99	0.84	1.00	0.11
	200	0.99	0.87	1.00	0.13
2	**Current Study Nobs**	**0.39**	**0.09**	**0.04**	**0.01**
	20	0.15	0.07	0.03	0.02
	40	0.42	0.23	0.03	0.04
	60	0.61	0.34	0.05	0.05
	80	0.72	0.46	0.07	0.06
	**100**	**0.83**	**0.55**	**0.05**	**0.05**
	120	0.89	0.63	0.05	0.06
	140	0.94	0.69	0.05	0.06
	160	0.96	0.75	0.05	0.07
	180	0.98	0.80	0.05	0.07
	200	0.99	0.83	0.06	0.08
3	**Current Study Nobs**	**0.05**	**0.08**	**0.07**	**0.02**
	20	0.04	0.17	0.19	0.05
	40	0.15	0.53	0.54	0.11
	60	0.25	0.77	0.78	0.16
	**80**	**0.40**	**0.90**	**0.91**	**0.22**
	100	0.50	0.95	0.95	0.25
	120	0.59	0.98	0.98	0.28
	140	0.65	0.99	0.99	0.34
	160	0.72	0.99	1.00	0.38
	180	0.79	1.00	1.00	0.43
	200	0.83	1.00	1.00	0.46
4	**Current Study Nobs**	**0.10**	**0.03**	**0.04**	**0.01**
	20	0.12	0.03	0.13	0.18
	40	0.48	0.09	0.37	0.47
	60	0.75	0.19	0.55	0.64
	**80**	**0.87**	**0.25**	**0.71**	**0.76**
	100	0.93	0.34	0.80	0.86
	120	0.98	0.40	0.86	0.92
	140	0.99	0.48	0.93	0.95
	160	0.99	0.53	0.95	0.96
	180	1.00	0.59	0.98	0.98
	200	1.00	0.63	0.98	0.99
5	**Current Study Nobs**	**0.02**	**0.33**	**0.25**	**0.03**
	20	0.02	0.68	0.40	0.05
	**40**	**0.04**	**0.98**	**0.81**	**0.10**
	60	0.03	1.00	0.94	0.13
	80	0.04	1.00	0.98	0.16
	100	0.04	1.00	1.00	0.15
	120	0.04	1.00	1.00	0.19
	140	0.05	1.00	1.00	0.21
	160	0.05	1.00	1.00	0.23
	180	0.06	1.00	1.00	0.26
	**200**	0.06	1.00	1.00	0.26
6	**Current Study Nobs**	**0.10**	**0.09**	**0.03**	**0.14**
	20	0.06	0.07	0.04	0.41
	40	0.14	0.17	0.08	0.92
	60	0.19	0.28	0.11	0.98
	80	0.25	0.38	0.14	1.00
	100	0.31	0.49	0.15	1.00
	120	0.35	0.57	0.19	1.00
	140	0.42	0.67	0.21	1.00
	160	0.46	0.72	0.26	1.00
	180	0.51	0.77	0.30	1.00
	**200**	**0.57**	**0.81**	**0.31**	**1.00**
7	**Current Study Nobs**	**0.17**	**0.19**	**0.04**	**0.02**
	20	0.07	0.25	0.03	0.03
	40	0.25	0.64	0.04	0.05
	**60**	**0.41**	**0.84**	**0.04**	**0.08**
	80	0.60	0.95	0.05	0.10
	100	0.71	0.98	0.05	0.10
	120	0.81	0.99	0.06	0.10
	140	0.86	1.00	0.05	0.13
	160	0.91	1.00	0.05	0.13
	180	0.93	1.00	0.05	0.14
	200	0.96	1.00	0.05	0.15
8	**Current Study Nobs**	**0.07**	**0.07**	**0.06**	**0.02**
	20	0.01	0.04	0.04	0.02
	40	0.05	0.10	0.10	0.03
	60	0.08	0.17	0.16	0.04
	80	0.11	0.22	0.22	0.04
	100	0.14	0.25	0.28	0.04
	120	0.17	0.30	0.31	0.05
	140	0.19	0.36	0.37	0.06
	160	0.20	0.39	0.42	0.07
	180	0.24	0.45	0.49	0.06
	200	0.26	0.51	0.54	0.07
10	**Current Study Nobs**	**0.15**	**0.03**	**0.06**	**0.01**
	20	0.14	0.03	0.11	0.03
	40	0.39	0.04	0.35	0.06
	60	0.57	0.04	0.55	0.09
	80	0.70	0.05	0.69	0.11
	**100**	**0.80**	**0.05**	**0.80**	**0.09**
	120	0.87	0.05	0.87	0.12
	140	0.92	0.05	0.92	0.13
	160	0.95	0.05	0.95	0.16
	180	0.97	0.04	0.96	0.16
	200	0.98	0.05	0.98	0.17
